# Geologic, seasonal, and atmospheric predictors of indoor home radon values

**DOI:** 10.1088/2752-5309/acdcb3

**Published:** 2023-06-21

**Authors:** Ellen J Hahn, William C Haneberg, Stacy R Stanifer, Kathy Rademacher, Jason Backus, Mary Kay Rayens

**Affiliations:** 1 BREATHE, College of Nursing, University of Kentucky, Lexington, KY, United States of America; 2 Kentucky Geological Survey, University of Kentucky, Lexington, KY, United States of America

**Keywords:** radon, indoors, bedrocks, soil

## Abstract

Exposure to tobacco smoke and radon cause lung cancer. Radioactive decay of naturally occurring uranium in bedrock produces radon. Seasonality, bedrock type, age of home, and topography have been associated with indoor radon, but the research is mixed. The study objective was to examine the relationships of geologic (soil radon and bedrock) and seasonal (warm and cold times of the year) factors with indoor home radon values in citizen scientists’ homes over time, controlling for atmospheric conditions, topography, age of home, and home exposure to tobacco smoke. We collected and analyzed indoor radon values, soil radon gas concentrations, and dwelling- and county-level geologic and atmospheric conditions on 66 properties in four rural counties during two seasons: (1) summer 2021 (*n* = 53); and (2) winter/spring 2022 (*n* = 52). Citizen scientists measured indoor radon using Airthings radon sensors, and outdoor temperature and rainfall. Geologists obtained soil radon measurements using RAD7 instruments at two locations (near the dwelling and farther away) at each dwelling, testing for associations of indoor radon values with soil values, bedrock type, topography, and atmospheric conditions. Bedrock type, near soil radon levels, home age, and barometric pressure were associated with indoor radon. Dwellings built on carbonate bedrock had indoor radon values that were 2.8 pCi/L (103.6 Bq m^−3^) higher, on average, compared to homes built on siliclastic rock. Homes with higher near soil radon and those built <40 ago were more likely to have indoor radon ⩾4.0 pCi/L (148 Bq m^−3^). With higher atmospheric barometric pressure during testing, observed indoor radon values were lower. Seasonality and topography were not associated with indoor radon level. Understanding relationships among bedrock type, soil radon, and indoor radon exposure allows the development of practical predictive models that may support pre-construction forecasting of indoor radon potential based on geologic factors.

## 1. Introduction

Exposure to radon is a known cause of lung cancer [1, 2] and several epidemiological studies implicate radon exposure in breast cancer [3], malignant melanoma [4], and pulmonary function in COPD patients [5]. One recent observational study (*N* = 68) demonstrated elevated inflammatory markers (C-reactive protein and interleukin-1*β*) in children and adolescents (ages 6–14 years old) exposed to radon [6]. The radioactive decay of naturally occurring uranium in bedrock produces radon. Specifically, dwellings built in areas underlain by phosphatic Ordovician age limestones, Mississippian age limestones, and Devonian age black shales in Kentucky tend to have higher indoor radon values than homes built on other kinds of bedrock [7, 8].

Indoor radon potential has historically been generalized using county-wide hazard ratings [9]; however, physical phenomena are not controlled by geopolitical boundaries and single-value county maps do not address within-county variation in radon potential. Haneberg *et al* [7] previously developed a robust predictive tool by spatially joining 28 years of observed home radon data (*N* = 70 000+) with a statewide 1:24 000-scale bedrock geologic map within a geographic information system (GIS) framework to create a statistically valid indoor radon potential map. County-level maps made from the statewide map and linking the percent of homes tested for radon and all aspects of radon risk were then used to develop within-county risk infographics [7]. These new maps tell a different story than the U.S. Environmental Protection Agency (EPA) radon maps. For example, Kentucky’s EPA map gives the false impression that radon exposure is low in rural KY. In contrast, the new intra-county maps show significant variability, with some rural areas having extremely high radon potential.

In a previous study of indoor radon and bedrock geology in Kentucky [7], the authors found that homes with the highest indoor radon potential were, in order of decreasing potential, built in areas underlain by Mississippian-age limestones containing little or no uranium, Ordovician-age limestones containing phosphatic strata with some uranium, and Devonian-age black shales that are typically high in uranium. Other than the black shale, siliclastic sedimentary rocks and sediments, in contrast, were associated with low indoor radon levels. Others have reported associations between bedrock lithology, mineralogy, geologic structures such as faults, and indoor radon measurements in different geologic settings and parts of the world [10–18].

Previous studies of radon in cave systems and homes built on karst terrain show location dependent correlations involving both seasonality and topography [19–22]. Other studies found no such seasonal correlation [23]. Human factors (e.g. heating, cooling, construction type) and atmospheric factors (e.g. outdoor air temperature, atmospheric pressure, relative humidity, soil moisture) may impact seasonality. Understanding the relationships between bedrock type and seasonality is critical in the development of practical predictive models that allow for pre-construction forecasting and mitigation of indoor radon potential based upon soil radon measurements or, more generally, bedrock type.

The purpose of the study was to investigate seasonal indoor radon variation across geographic regions in four rural Kentucky counties with different bedrock geology. We aimed to examine the relationships of geologic (soil radon and bedrock) and seasonal (warm and cold times of the year) factors with indoor home radon values in citizen scientists’ homes over time, controlling for atmospheric conditions, topography, age of home, and home exposure to tobacco smoke.

## 2. Method

**Figure 1.**
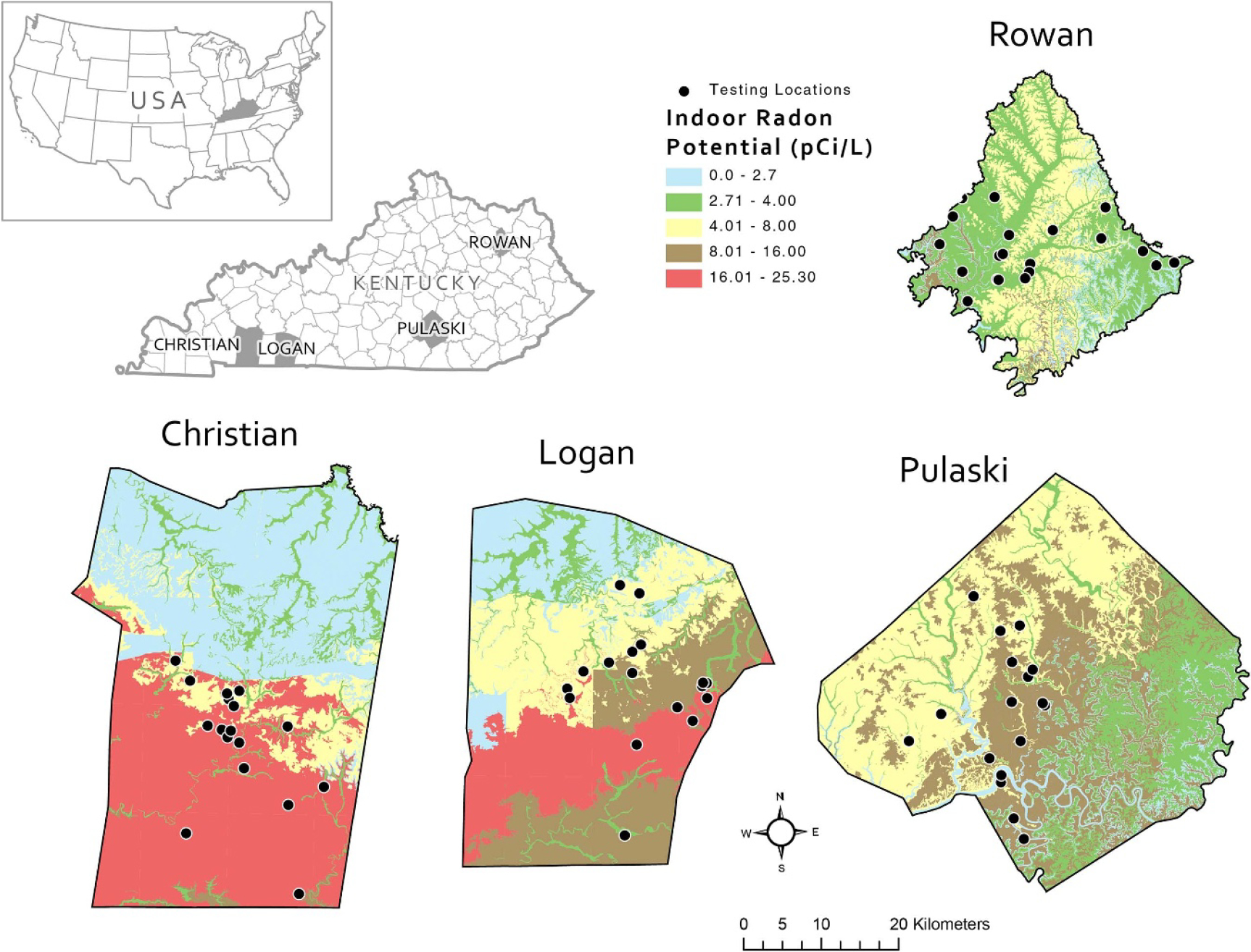
Maps showing the four counties with indoor radon potential distributions in pCi/L from the geologically based Kentucky indoor radon potential map [7]. The black dots represent testing locations in each county

This study is part of a larger community-engaged research project, RADAR, which stands for Residents Acting to Detect and Alleviate Radon. The overall project goal is to increase access to radon testing and affordable radon mitigation in rural communities using geohealth methodology and a citizen science approach. We selected four rural counties in Kentucky using purposive sampling; in each county, we recruited a convenience sample of at least 15 homeowners and trained them as citizen scientists to test their homes for radon using digital radon technology, Airthings Corentium Home detectors. The four rural counties varied in indoor radon potential, and we matched counties by population size and socioeconomic status (figure 1).

**Figure 2.**
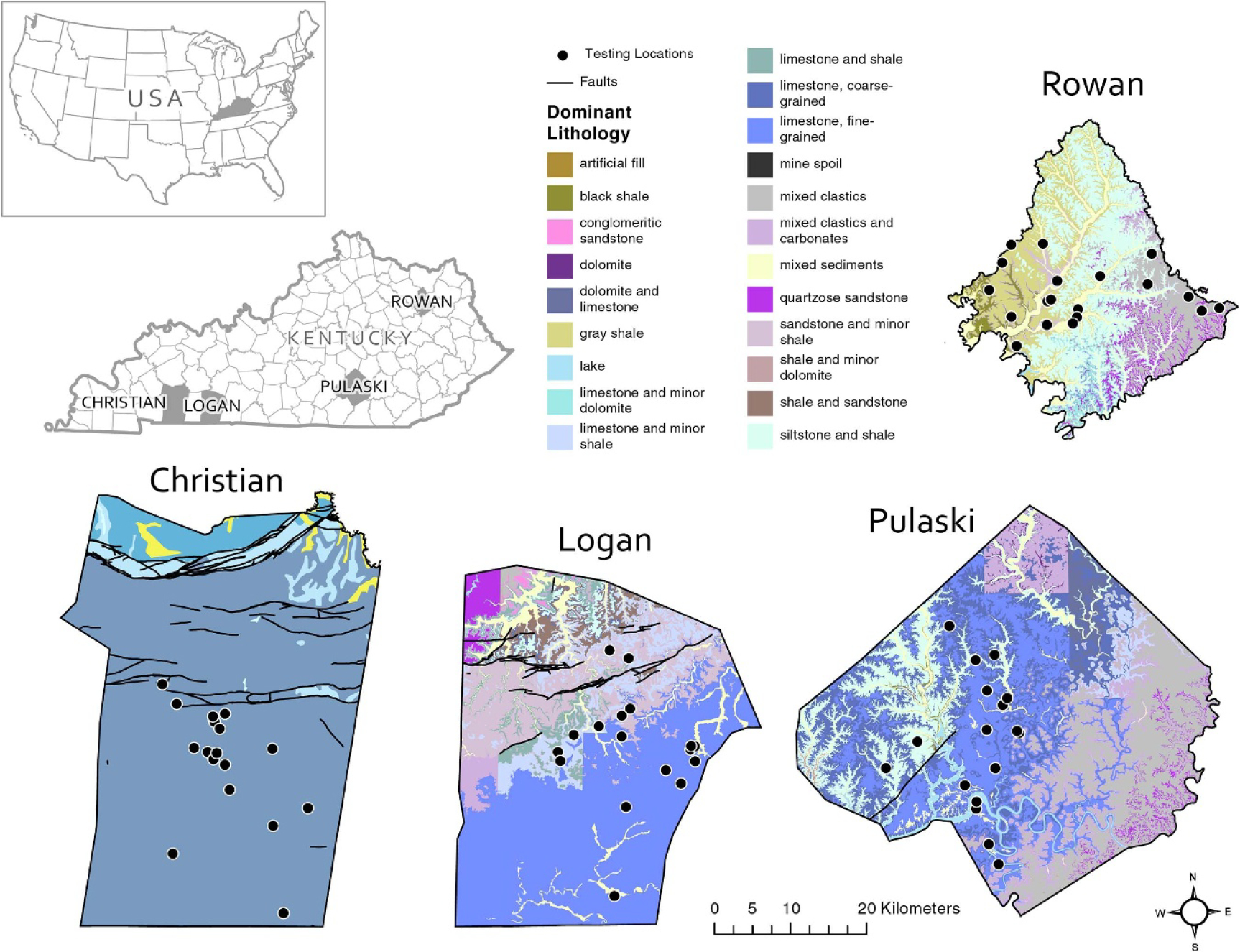
Maps showing bedrock type in the four study counties, labeled according to the dominant lithology attribute of the Kentucky digital geologic map (https://kgs.uky.edu/kygeode/geomap/?layoutid=3).

Figure 2 illustrates that carbonate sedimentary rocks (primarily limestone) with smaller areas underlain by siliclastic sedimentary rocks such as sandstone, siltstone, and shale underlie sizeable portions of three of the study counties (Christian, Logan, and Pulaski). Siliclastic sedimentary rocks underlie Rowan County. Also shown in figure 2, complex fault systems cut across Christian and Logan counties, whereas Pulaski County has only one mapped fault and Rowan County has none. Carbonate rock is soluble and easily dissolved in Kentucky’s humid subtropical climate, giving rise to gently rolling karst landscapes that stand in contrast to the rougher and steeper landscapes developed in areas underlain by siliclastic rock. This nexus between bedrock type and landscape is responsible for a concentration of homes (thus, our testing locations) in areas of gentle topography that are underlain by carbonate rock and more suitable for development than rougher parts of the counties underlain by siliclastic bedrock. We describe county sample selection, sampling citizen scientists and training elsewhere [24], as well as the RADAR outcomes of using a citizen science approach and Airthings to advance radon testing in the larger research study.

### 2.1. Study design and population

We used a prospective, non-experimental design to collect and analyze indoor radon values, soil radon gas concentrations, and dwelling- and county-level atmospheric conditions on 73 dwellings in the four rural study counties during two seasons. We could only retain 66 dwellings for this analysis; homeowners had mitigated for radon in the other 7 dwellings. Of the 66 dwellings, we could retain 62 in the time series models because the data for the other 4 homes were incomplete for one or more environmental factors. The two testing periods were: (1) summer 2021 (mid-June through mid-August; *n* = 53); and (2) winter/spring 2022 (late February through early May; *n* = 52). Our intention was to evaluate seasonality by having one testing period during warm summer months and the second testing period during chilly winter months. However, we delayed the start of the fieldwork in early 2022 due to a surge in COVID-19 infections and low vaccination rates in the study counties. Moving the winter testing start date to late February posed conflicts with local school district spring breaks, which could have reduced citizen scientist participation, extending winter testing into early May. Therefore, we refer to the second testing period as winter/spring.

Citizen scientists deployed Airthings Corentium Home detectors in their homes for two 14 d indoor radon testing periods. During the home radon testing period, citizen scientists each collected daily outdoor air temperature and rainfall amounts at their properties. They also documented daily if they kept closed home conditions (e.g. windows and exterior doors closed; yes or no) most of the day. During the home radon testing period, a team of two geologists visited each dwelling and collected two soil radon gas samples in the yard. The geologists also documented weather conditions at the county level on the date of soil radon gas measurements. A total of 34 of the 66 citizen scientists included in this analysis participated in both testing periods in summer 2021 and winter/spring 2022 and had complete data for environmental factors and exposure to tobacco smoke in the home; the other 32 participated at one of the two testing periods. We recruited and enrolled citizen scientists using an informed consent protocol approved by the Institutional Review Board.

### 2.2. Methods to measure or estimate exposures and covariates

We measured soil radon gas using Durridge RAD7 radon detectors, which are continuous electronic radon detectors with real-time monitoring, spectral analysis, and typically capable of obtaining measurements in two hours or less over a range of 0.1 pCi/L–20 000 pCi/L (1 pCi/L = 37 Bq m^−3^; pCi/L is the customary unit of measurement in the study areas). Kentucky Geological Survey geologists measured soil radon gas concentrations at each citizen scientist’s home using a standardized fieldwork protocol and site visit information sheets during the two 14 d indoor radon testing periods. We selected two sample locations during each visit, one approximately 2 m from the dwelling and the second approximately 3 m from the home, and we noted both locations on the site visit information sheet. Geologists pushed or gently drove the sampling probe to a depth of 0.3 m–0.6 m based on the amount of resistance encountered, and they took care not to damage the probe. They then measured soil radon gas using a purge-sniff-grab procedure consistent with the instrument manufacturer’s instructions. Soil gas radon has long been known to vary with respect to location, depth, and soil moisture conditions [25–28]; our measurements during each of the two testing periods represent snapshots of conditions at those depths and locations.

We supplied analog rain gauges and outdoor thermometers to the trained citizen scientists and asked them to report rainfall (in inches) and air temperature (°F) at their dwellings daily for both 14 d indoor radon testing periods. We asked citizen scientists to document on each day of testing whether the home had any windows or doors open. Additionally, the geologists obtained mean daily barometric pressure (mb) at the county level from the meteorological station with publicly accessible data nearest to each citizen scientist location using the WeatherData function in the computer program Mathematica [29]. The county level data were for the day on which we obtained soil radon measurements at each location.

To understand the effect of topography on soil and indoor radon variables, we calculated values for three key topographic variables at each citizen scientist’s home: slope angle, topographic roughness, and topographic position index (TPI) based upon a version of the publicly available statewide Kentucky From Above (https://kyfromabove.ky.gov) airborne lidar digital elevation model (DEM) aggregated from an original cell size of approximately 1.5 m (original units: 5 ft) to a cell size of approximately 7.6 m (original units: 25 ft) to reduce the effects of highly localized topographic variations. We calculated slope angle using the standard slope function in ArcGIS Pro, which fits a plane passing through a specified DEM cell and its eight immediate neighbors. The 101-cell value was selected based on a heuristic consideration of typical ridge-to-ridge and valley-to-valley distances across much of Kentucky to ensure that the full range of local topographic variability was included in the TPI calculations [30].

We operationalized exposure to tobacco smoke in the home by asking citizen scientists two questions to determine if anyone living in the home (including themselves) used combustible tobacco products and/or e-cigarettes or other vaping products. If respondents answered yes to either question, we coded this as tobacco smoke exposure in the home [31]. Participants reported the age of their home (i.e. year built), and we subtracted this from the year they completed their first survey to determine age of dwelling. Given the right-skewed distribution of this measure, we used a median split to create an indicator for homes built in the last 40 years.

### 2.3. Outcome definition and measurements

We used Airthings digital radon monitors to estimate 2 week average radon values in picocuries per liter of air (pCi/L). The Airthings (Corentium Home model) is a portable battery-operated radon gas detector with auto-calibration that is easy to operate, and citizen scientists find it easy to use. The device has a small monitor that tracks real-time radon values in pCi/L over extended periods and provides an average value over the past 24 h. The device displays both long-term and short-term readings. The short-term average alternates between displaying radon values for the last day (updated hourly) and for the last 7 d (updated once a day). The long-term average represents the average radon value for the ongoing measurement, in this case 2 weeks (updated once a day). Citizen scientists used the Airthings measurement device during this phase as well as the earlier phase of the study [24].

### 2.4. Statistical analyses

Since the distributions of radon values, whether obtained indoors or from the soil outside dwellings, was positively skewed, we used a log transformation so the values would better approximate a normal distribution. To ensure definition of the transformed values, we added a constant of 0.1 to each of the observed radon assessments prior to transformation. We summarized all study variables descriptively using means, standard deviations, and ranges, or frequency distributions, as appropriate. We used the coefficient of variation to identify the degree of variability relative to the mean of each of the continuous variables.

To evaluate the environmental factors that were most strongly associated with indoor radon, we used two methods appropriate for repeated measures modeling, with time as the repeated factor. For the continuous outcome of log-transformed indoor radon, we used multivariable linear repeated measures mixed modeling. For the binary outcome of high indoor radon (as indicated by a value of ⩾4 pCi/L), we used multivariable generalized estimating equations (GEEs) modeling, which is an extension of logistic regression for more than one timepoint. Since outdoor temperatures and season of testing were related (i.e. there was a significant difference in average temperatures between the two timepoints), we could not include these values in the analysis. Rather, we retained time/season as it is a key variable in the repeated measures modeling.

Both repeated measures strategies include the participants who did not complete both assessments in the analysis, assuming the missing data were missing at random. This is relevant since some citizen scientists only completed the Time 1 assessment (*n* = 53) and withdrew from the study, while we recruited others as replacements for the larger study and only completed the Time 2 assessment (*n* = 52). Slightly more than half of participants included in this analysis completed both assessments (52%). There were no differences in any demographic factors between those who completed both assessments and those who completed only one, so the assumption of missing at random is reasonable. As an evaluation of the presence of multicollinearity in the model, we reviewed the variance inflation factors for the variables in the models; given they were all less than 1.7, there is little evidence of parameter distortion due to associations among the independent variables. We conducted all analyses using SAS, v. 9.4; we used an alpha level of .05 for inferential testing.

## 3. Results

A sample of 66 citizen scientists forms the basis of this analysis. Seven additional citizen scientists participated but reported home radon mitigation prior to the geo-assessment portion of the study. Since the focus of this analysis is on environmental factors associated with indoor radon, we omitted those who had mitigated for radon from this analysis.

The average age of the 66 participants was 51 (SD = 13), and the citizen scientists ranged from 25 to 78 years; most participants identified their gender as female (69%; table 1). Most citizen scientists were White, non-Hispanic (89%), with the remainder indicating they were Black or African American (and non-Hispanic). Two-thirds completed at least a 4 year college degree (67%), and slightly more than half reported a household income at or above $75 000 (52%). Very few participants used tobacco products (14%); the most common products used were cigarettes and e-cigarettes. Nearly one-fourth had a family history of lung cancer (21%).

A descriptive summary of the environmental variables, including indoor and outdoor radon (all log-transformed), having one or more smokers and/or e-cigarette users living in the home, age of home, the 14 d averages of precipitation and barometric pressure, and an indicator for whether the dwelling is in an area underlain by carbonate bedrock (primarily limestone versus the alternative of siliclastic sandstone, siltstone, shale, or alluvium) is shown in table 2. While 66 participants completed data collection for at least one timepoint, the number with measurements at each timepoint varied; 53 participated at Time 1 (summer 2021) and 52 at Time 2 (winter/spring 2022). At Time 1, 15% of participants had a high indoor radon value of ⩾4 pCi/L, and this increased slightly to 22% at Time 2. One-quarter (24%) of the citizen scientists indicated they lived in a household with one or more persons who used combustible tobacco products (cigarettes, cigars, or pipes) and/or electronic smoking devices (e-cigarettes, e-cigars, e-pipes). Nearly half had a home built in the last 40 years (49%). Two-thirds of participants lived in a dwelling built above carbonate bedrock (64%).

There was low variability among observed values for barometric pressure and log-transformed soil radon values at each timepoint. The coefficients of variation (CVs) were equal to 0.6% (Time 1) and 0.8% (Time 2) for barometric pressure. At Time 1, soil radon CVs were 27.2% (near the dwelling) and 39.6% (far from the home). At Time 2, the CVs for soil radon were smaller, specifically 14.9% and 16.7% for near and far away from the dwelling, respectively. There was greater variability among the measures of log-transformed indoor radon and 14 d precipitation at each timepoint, with CVs for indoor radon equal to 206.5% (Time 1) and 200.0% (Time 2) and precipitation CVs of 82.4% (Time 1) and 75.0% (Time 2). The CVs for the time-invariant topographic variables ranged from 53.7% for the TPI to 94.3% and 94.8% for topographic roughness and slope angle, respectively. Between Time 1 and Time 2, radon measurements, whether indoor or outdoor, tended to be higher at Time 2 (winter/spring) compared with Time 1 (summer). Barometric pressure was unchanged between Time 1 and Time 2. Precipitation was slightly greater at Time 2 (winter/spring), consistent with historical precipitation patterns, though the summer (i.e. Time 1) exhibited a greater range of observed 2 week precipitation values. Regardless of whether the testing period was Time 1 or Time 2, citizen scientists reported closed home conditions (with no open doors/windows) for over 99% of all days reported (i.e. up to 14 d per testing period for each of the 66 participants).

## 4. Repeated measures modeling

The linear repeated measures mixed model was significant overall (*χ*^*2*^ = 10.6, *p* = .0012). The variables significantly associated with the log-transformed continuous indoor radon value in this model included the indicator for the dwelling built <40 ago, 14 d barometric pressure, and the presence of carbonate bedrock below the house (table 3; figure 2). Homes built in the last 40 years and those above carbonate bedrock typically had higher indoor radon levels relative to older homes and those built over siliclastic bedrock. A higher average barometric pressure was associated with lower radon in the home.

In the repeated measures GEEs model to identify predictors of whether the home had a radon level that was at or above 4 pCi/L, the significant predictors included the indicator for age of home built in the last 40 years, 14 d barometric pressure, the carbonate bedrock indicator, and log-transformed soil radon near the dwelling. Compared to older dwellings, if a home was built <40 years ago, the odds of a high indoor radon level increased by over 3000% (odds ratio (OR) = 34.90). Consistent with the linear model, higher barometric pressure was associated with a decreased likelihood of high indoor radon. For each 1-point increase in barometric pressure, the likelihood of having an indoor radon measure of 4 pCi/L or above decreased by 18%. Conversely, homes built above carbonate bedrock had an increased likelihood of high indoor radon of over 2000% (OR = 23.59), compared to homes built over siliclastic bedrock. Similarly, increased soil radon close to the home was associated with an elevated likelihood of high indoor radon. For each 1-point increase in the log-transformed near-dwelling soil radon, the odds of an indoor radon level of at least 4.0 pCi/L increased by 134%.

Given the typical ranges of values for barometric pressure and soil radon, it is reasonable to estimate the increase or decrease in odds associated with larger changes in each. For each 5-point increase in barometric pressure, the odds of a high indoor radon measurement of 4 pCi/L or above decreased by 62% (OR = 0.38; 95% confidence interval (CI): 0.17–0.85). For each 2-point increase in soil radon near the dwelling, the odds of a high indoor radon assessment increased by 450% (OR = 5.49; 95% CI: 1.26–23.99). Other environmental factors were not significant in the model.

## 5. Conclusion

We found that bedrock type, age of the home, soil radon levels near the dwelling, and barometric pressure were associated with indoor radon value. Dwellings built on carbonate bedrock had indoor radon values that were 2.8 pCi/L (103.6 Bq m^−3^) higher, on average, compared to homes built on siliclastic rock. Similarly, homes underlain by carbonate bedrock were more than 2000% more likely to have high indoor radon (⩾4 pCi/L). Homes built less than 40 years ago had an average indoor radon value that was 1.9 pCi/L (70.3 Bq m^−3^) higher than the older homes in the study. Consistent with this, dwellings less than 40 years old were more than 3000% more likely to have a radon level ⩾4 pCi/L. For each 2-unit increase in soil radon level, the home was more than 400% more likely to have indoor radon ⩾4.0 pCi/L. Higher atmospheric barometric pressure during testing was associated with lower indoor radon exposure and lower likelihood of having an observed indoor radon value at or above 4 pCi/L.

Our finding that homes built over carbonate bedrock—most commonly limestone—have higher indoor radon potential than those built over siliclastic bedrock such as sandstone, siltstone, or shale, or unlithified siliclastic alluvium, is consistent with the results reported by Haneberg *et al* [7]. In contrast to some Kentucky limestone containing layers rich in the calcium phosphate mineral apatite, which can accommodate uranium as an impurity in its crystal structure, most of the limestone underlying the homes described in this paper is low in uranium that could give rise to radon. Geologic processes that might explain high indoor radon results in homes built above low uranium limestone include [1]: residual accumulation of insoluble uranium-bearing minerals in soils, as soluble carbonate minerals may be dissolved during chemical weathering of the limestone [2]; waterborne transport and subsequent deposition of uranium-bearing minerals in the innumerable open fractures and cave conduits are characteristic of these limestones; or [3] airborne transport of radon from deeper uranium-rich bedrock layers. We need more research to explain the role of bedrock and other geological factors (e.g. proximity to faults or sinkholes) in influencing indoor radon concentrations, given our finding that carbonate bedrock, low in uranium, is associated with high indoor radon values.

We found that homes built <40 years ago were more likely to have elevated indoor radon. Similar to our findings, Brookins [32] concluded that newer homes in New Mexico, which had refrigerated air conditioning, had higher indoor radon concentrations. However, the homes with high radon also tended to be closer to or built on granite and limestone bedrock with moderately high uranium levels exposed in nearby mountains. One explanation for newer homes having higher radon may be the use of energy efficiency building standards linked to higher indoor radon [33]. As builders design energy efficient homes for air tightness, radon can enter through cracks in foundations and become trapped; whereas older homes constructed without energy-efficient standards are more likely to have gaps and cracks where radon can escape. Use of different building materials may be another reason that age of home is associated with indoor radon level. Barros-Dios *et al* [34] found older homes were primarily constructed of stone in both the exterior and interior of the home and they had significantly higher indoor radon levels, followed by stone-and-brick, and brick alone. Yazzie *et al* [35] found that homes constructed of cement and wood and homes constructed of concrete and cement had higher mean indoor radon concentrations than mobile homes and those constructed of wood and cement and wood alone. We need further study to examine the association of home building materials, age of home, and indoor radon levels. Regardless of the reason, the fact that newer homes may be more likely to expose residents to radon creates disparities as occupants of newer homes tend to be the youngest and most vulnerable to radon exposure (i.e. children and pregnant women) [36].

We also found that soil radon values near the homes were related to indoor radon levels in the homes but that soil radon measurements slightly farther away obtained at the same time were not. Previous studies have shown that both soil radon and indoor radon levels are spatially correlated, meaning that locations close to another are more likely to have similar values than locations farther apart [37–41], as are many other soil properties [42]. In that regard, it is not unexpected that near-home soil radon values are correlated with in-home radon values.

Our finding that higher barometric pressure is associated with lower indoor radon is similar to those of three studies [43–45], in which indoor radon values were negatively correlated with outdoor barometric pressure. Chen *et al* [45] developed a field-validated advective-diffusive soil radon transport model to evaluate the effects of cyclic barometric pressure fluctuations on soil radon concentrations. They showed that barometric pressure fluctuations at the ground surface reduced soil radon concentrations at shallow depths during periods of high barometric pressure and increased soil radon concentrations during periods of low barometric pressure in a manner consistent with an advectively dominated air-pumping process. Their findings are consistent with our observed association between lower indoor radon levels and high barometric pressures. However, the research literature on the effects of barometric pressure on indoor radon is mixed. Kitto [46] and Keskikuru *et al* [47] found no correlation between barometric pressure and indoor radon concentrations. One study [48] reported a positive correlation, and another study [49] found a seasonal effect, with a positive relationship in the winter but a negative correlation in summer months. The relationship between barometric pressure and indoor radon may be complicated by the weather conditions, age of the home, the type of heating, ventilation, and air conditioning (HVAC) system, and residential characteristics including building materials and the number of stories in the home [34].

Although other studies report an association between seasonality and indoor radon concentrations [32, 50–54], time of year did not predict indoor radon values in our study. Brookins [32] found that radon concentrations in homes in the Albuquerque, New Mexico area with refrigerated air conditioning did not show the same seasonal radon variability (high in winter and low in summer) as did homes without refrigerated air conditioning (e.g. those using evaporative coolers that provide steady ventilation with outside air). Consistent with EPA home radon testing recommendations [55], the citizen scientists in the study reported here were asked to keep windows and exterior doors closed as much as possible when testing for indoor radon and, indeed, very few reported opening windows or doors on any of the days during either of the two testing periods. Maintaining closed home conditions during both 14 day testing periods may have contributed to the diminished effect of seasonality on indoor radon values. Additionally, our delay in beginning the winter measurements due, in part to the COVID-19 pandemic may have diminished expected differences between summer and winter/spring in this study.

Our research did not find a significant correlation between precipitation and indoor radon levels. Previous research has shown a complicated relationship between indoor radon levels and soil moisture or precipitation. Some studies have reported an association between precipitation or soil moisture and increased indoor radon levels [56–58], whereas others have reported minimal or no correlation between rainfall and indoor radon values [43, 46, 59]. In a study of soil radon levels, Sundal *et al* [60] found associations between soil radon and both temperature and barometric pressure, but not precipitation or soil moisture.

Our geologic and residential findings, specifically related to age of home, carbonate bedrock, and soil radon have public policy implications. The use of radon resistant new construction (RRNC) techniques ensures new homes are built to minimize radon intrusion, yet very few states have adopted RRNC policies [61]. Some states require RRNC for all new construction while others specify its use only in high radon risk zones. The National Radon Action Plan 2021–2025, which is supported by the Environmental Protection Agency and American Lung Association among others, calls for ‘building in’ risk reduction through state and local building codes that promote radon control at the time of construction [62]. In Kentucky, no such building codes exist. Our findings that newer homes and low-uranium carbonate bedrock are associated with high indoor radon support policies that recommend or require RRNC building codes in new homes and in areas underlain by carbonate bedrock. Furthermore, we recommend that policymakers and environmental health professionals reevaluate traditional methods of radon hazard assessment based on bedrock or soil uranium content.

### 5.1. Strengths and limitations

One of the strengths of this study is the inclusion of citizen scientists as research partners. Citizen scientists collected and reported daily residential radon, temperature, and rainfall data at their property. In doing so, the citizen scientists contributed to the generation of new knowledge related to radon intrusion in homes. Data collection of this kind may have been otherwise difficult for researchers alone due to lack of access to individual properties, constraints on time, planning, and other resources. Citizen scientists’ use of the Airthings Corentium Home, which provides real-time continuous radon monitoring, in conjunction with simultaneous collection of outdoor geologic and atmospheric factors is a novel approach to evaluating radon risk status. One limitation of the Airthings Corentium, however, is that it does not measure indoor temperature or barometric pressure, only radon.

The primary limitation of this study is sample size. While we selected the number of participants *a priori* to guarantee sufficient power for detection of prespecified effects, a slightly larger sample may have uncovered additional relationships not established here. Another limitation is that the winter/spring testing period included warmer weather in the spring months (e.g. daily elevated temperatures above 80°F in late April and early May 2022). Further, we did not measure ventilation or type of HVAC system inside the dwellings which could impact indoor radon values regardless of (or in addition to) atmospheric pressure. We also asked citizen scientists to keep their homes closed during both testing periods and this could have diminished the seasonal effects if they normally would have opened doors and windows during the warmer months. In addition, we did not measure primary (e.g. grain size, thickness, and mineral composition) and secondary (e.g. permeability, moisture content) soil properties which could impact soil gas and indoor radon concentrations. Nor did we measure internal building materials or stories of the home [34]. Lastly, measuring radon in water was beyond the scope of this study as nearly all participants were on public water supplies; well water can be a source of radon exposure [63]. Future studies in this area will benefit from larger sample sizes, better separation between winter and summer testing periods, consideration of both soil and bedrock properties, and consideration of housing characteristics including ventilation or type of HVAC system, building materials, water sources, and stories of the home.

In summary, we found that bedrock type, age of home, near soil radon levels, and barometric pressure were associated with indoor radon. Dwellings built on carbonate bedrock (low in uranium) had indoor radon values that were 2.8 pCi/L higher, on average, compared to homes built on siliclastic rock. Homes built less than 40 years ago had average radon levels that were 1.9 pCi/L higher than older homes. For each 2-unit increase in soil radon level, the home was more than 200% more likely to have indoor radon ⩾4.0 pCi/L. When the atmospheric barometric pressure was higher during testing, observed indoor radon values tended to be lower. Seasonality and topography were not associated with indoor radon level. Understanding the relationships among bedrock type, age of home, soil radon, and indoor radon exposure allows the development of practical predictive models that may support pre-construction forecasting of indoor radon potential based on geologic factors and may guide radon risk reduction policies such as RRNC, particularly in conjunction with energy efficiency goals, which may trap radon indoors without modifying building codes.

## Figures and Tables

**Table 1.** Demographic characteristics of citizen scientists (*N* = 66).

Variable	Mean (SD); range or *n* (%)

Age	51.0 (13.4); 25–78
Female gender	45 (69.2%)
White, non-Hispanic	58 (89.2%)
Completed college/graduate degree	44 (66.7%)
Household income ⩾$75 000	33 (51.6%)
Current tobacco user	9 (13.6%)
Family history of lung cancer	14 (21.2%)

**Table 2.** Descriptive summary of environmental factors for each dwelling at each timepoint (*N* = 66).

Variable	Time 1 (summer) (*n* = 53)	Time 2 (winter/spring) (*n* = 52)
	Mean (SD); range or *n* (%)	Mean (*SD*); range or *n* (%)

Log-transformed indoor radon (pCi/L)	0.46 (0.95); −1.46–3.66	0.60 (1.20); −2.30–3.75
Indoor radon ⩾ 4 pCi/L	8 (15.4%)	11 (21.6%)
Tobacco user(s) in the home	16 (24.2%)	16 (24.2%)
Home built <40 years ago	32 (48.5%)	32 (48.5%)
14 d precipitation (inches)	1.7 (1.4); 0–6.2	2.0 (1.5); 0–5.1
14 d barometric pressure (mb)	991.9 (5.5); 981.2–999.0	993.9 (8.2); 980.6–1007.4
Carbonate bedrock	42 (63.6%)	42 (63.6%)
Log-transformed near soil radon (pCi/L)	5.58 (1.52); 0.095–7.74	6.10 (0.91); 3.00–7.77
Log-transformed far soil radon (pCi/L)	4.95 (1.96); −2.30–7.80	6.00 (1.00); 3.61–7.64
Slope angle (degrees)	3.09 (2.93); 0.39–16.96	3.09 (2.93); 0.39–16.96
Topographic roughness (±degrees)	2.30 (2.17); 0.47–11.52	2.30 (2.17); 0.47–11.52
Topographic position index (0 ⩽ TPI ⩽ 1, dimensionless)	0.54 (0.29); 0.052–0.98	0.54 (0.29); 0.052–0.98

SD: standard deviation. 1 pCi/L = 37 Bq m^−3^; pCi/L is the customary unit of measurement in the study areas.

*Note*: Invariant between Time 1 and Time 2 either because we asked it only on initial survey or it is not subject to change; reflects all 66 participants who participated in one or both timepoints.

**Table 3.** Multivariable linear and logistic repeated measures models to identify dwelling-specific values that are predictive of level of indoor radon (*n* = 62).

Variable	Linear repeated measure mixed model		Generalized estimating equations model with repeated measures		
	Estimate (std. error)	*F* (*p*-value)	Estimate (std. error)	|Z| (*p*-value)	Odds ratio (OR) and (95% CI^a^ for OR)

Time	−0.138 (0.167)	0.7 (.41)	−0.575 (0.631)	0.9 (.36)	—
Tobacco users in the home	−0.149 (0.286)	0.3 (.60)	0.239 (0.944)	0.3 (.80)	1.27 (0.20–8.07)
Home built <40 years ago	**0.629 (0.263)**	**5.7 (.020)**	**3.553 (1.680)**	**2.1 (.034)**	**34.90 (1.30–938.51)**
14 d precipitation	0.0540 (0.0649)	0.7 (.41)	0.130 (0.210)	0.6 (.54)	1.14 (0.75–1.72)
14 d barometric pressure	**−.0324 (.0158)**	**4.2 (.043)**	**−0.195 (0.0822)**	**2.4 (.018)**	**0.82 (0.70–0.97)**
Carbonate bedrock	**1.019 (0.286)**	**12.7 (<.001)**	**3.161 (1.170)**	**2.7 (.0069)**	**23.59 (2.38–233.43)**
Near soil radon	0.103 (0.0818)	1.6 (.21)	**0.852 (0.376)**	**2.3 (.024)**	**2.34 (1.12–4.90)**
Far soil radon	0.115 (0.0862)	1.8 (.18)	0.515 (0.328)	1.6 (.12)	1.67 (0.88–3.19)
Slope	8.29x10^−3^ (0.0450)	<0.1 (.85)	0.0341 (0.115)	0.3 (.77)	1.03 (0.83–1.30)
Roughness	0.0140 (0.0655)	0.1 (.83)	0.0696 (0.214)	0.3 (.75)	1.07 (0.70–1.63)
TPI^b^	−0.403 (0.541)	0.6 (.46)	−1.682 (1.998)	0.8 (.40)	0.19 (0.0037–9.35)

a CI: confidence intervals.

b TPI: topographic position index.

*Note:* Bold reflects significant associations with level of indoor radon.

## Data Availability

All data that support the findings of this study are included within the article (and any supplementary information files). Any further distribution of this work must maintain attribution to the author(s) and the title of the work, journal citation and DOI.

## References

[1] Darby S 2005 Radon in homes and risk of lung cancer: collaborative analysis of individual data from 13 European case-control studies Br. Med. J. 330 223 10.1136/bmj.38308.477650.63 PMC546066 15613366

[2] Krewski D 2006 A combined analysis of North American case-control studies of residential radon and lung cancer J. Toxicol. Environ. Health A 69 533–97 16608828 10.1080/15287390500260945

[3] VoPham T, DuPre N, Tamimi RM, James P, Bertrand KA, Vieira V, Laden F and Hart JE 2017 Environmental radon exposure and breast cancer risk in the Nurses’ Health Study II Environ. Health 16 97 28882148 10.1186/s12940-017-0305-6 PMC5590193

[4] Boz S, Berlin C, Kwiatkowski M, Bochud M, Bulliard J-L, Zwahlen M, Röösli M and Vienneau D 2022 A prospective cohort analysis of residential radon and UV exposures and malignant melanoma mortality in the Swiss population Environ. Int 169 107437 10.1016/j.envint.2022.107437 36152363

[5] Wang VA, Koutrakis P, Li L, Liu M, Vieira CLZ, Coull BA, Maher EF, Kang C-M and Garshick E 2023 Particle radioactivity from radon decay products and reduced pulmonary function among chronic obstructive pulmonary disease patients Environ. Res. 216 114492 10.1016/j.envres.2022.114492 PMC9701170 36209792

[6] Taylor BK, Smith OV and Miller GE 2023 Chronic home radon exposure is associated with higher inflammatory biomarker concentrations in children and adolescents Int. J. Environ. Res. Public Health 20 246 10.3390/ijerph20010246 PMC9819293 36612568

[7] Haneberg WC, Wiggins A, Curl DC, Greb SF, Andrews WM Jr, Rademacher K, Rayens MK and Hahn EJ 2020 A geologically based indoor-radon potential map of Kentucky Geohealth 4 e2020GH000263 10.1029/2020GH000263 PMC7682569 33283125

[8] Hahn EJ, Gokun Y, Andrews Jr W M, Overfield BL, Robertson H, Wiggins A and Rayens MK 2015 Radon potential, geologic formations, and lung cancer risk Prev. Med. Rep. 2 342–6 10.1016/j.pmedr.2015.04.009 PMC4721325 26844090

[9] U.S. Environmental Protection Agency 1993 EPA map of radon zones–national summary Report No.: 402-R-93–071 Contract No.: 6604J (Washington, DC Environmental Protection Agency)

[10] Choubey VM, Sharma KK and Ramola R 1997 Geology of radon occurrence around Jari in Parvati Valley, Himachal Pradesh, India J. Environ. Radioact. 34 139–47

[11] Sundal AV, Henriksen H, Soldal O and Strand T 2004 The influence of geological factors on indoor radon concentrations in Norway Sci. Total Environ. 328 41–53 15207572 10.1016/j.scitotenv.2004.02.011

[12] Minda M, Tóth G, Horváth I, Barnet I, Hámori K and Tóth E 2009 Indoor radon mapping and its relation to geology in Hungary Environ. Geol. 57 601–9

[13] Borgoni R, De Francesco D, De Bartolo D and Tzavidis N 2014 Hierarchical modeling of indoor radon concentration: how much do geology and building factors matter? J. Environ. Radioact. 138 227–37 25261869 10.1016/j.jenvrad.2014.08.022

[14] Friedmann H 2017 Indoor radon, geogenic radon surrogates and geology–Investigations on their correlation J. Environ. Radioact. 166 382–9 27158059 10.1016/j.jenvrad.2016.04.028

[15] Dai D, Neal FB, Diem J, Deocampo DM, Stauber C and Dignam T 2019 Confluent impact of housing and geology on indoor radon concentrations in Atlanta, Georgia, United States Sci. Total Environ. 668 500–11 30852225 10.1016/j.scitotenv.2019.02.257 PMC6456363

[16] Floriă Ş 2020 The path from geology to indoor radon Environ. Geochem. Health 42 2655–65 31897872 10.1007/s10653-019-00496-z

[17] Thumvijit T, Chanyotha S, Sriburee S, Hongsriti P, Tapanya M, Kranrod C and Tokonami S 2020 Identifying indoor radon sources in pa Miang, Chiang Mai, Thailand Sci. Rep. 10 17723 10.1038/s41598-020-74721-6 PMC7576592 33082391

[18] Banríon M, Elío J and Crowley Q 2022 Using geogenic radon potential to assess radon priority area designation, a case study around Castle island, Co Kerry, Ireland J. Environ. Radioact. 251 106956 10.1016/j.jenvrad.2022.106956 35780671

[19] Gammage RB, Dudney CS, Wilson DL, Saultz RJ and Bauer BC 1992 Subterranean transport of radon and elevated indoor radon in hilly karst terrains Atmos. Environ. A 26 2237–46

[20] Dudney C, Hawthorne A, Wilson D and Gammage R 1992 Indoor 222Rn in Tennessee Valley houses: seasonal, building, and geological factors Indoor Air 2 32–39

[21] Medici F and Rybach L 1994 Measurements of indoor radon concentrations and assessment of radiation exposure J. Appl. Geophys. 31 153–63

[22] Hughes JR, Turk B and Cardwell R (eds) 1998 Karst geology, radon fluctuations and implications for measurement and mitigation. Int. Radon Symp. (Athens: SRRTC)

[23] Pilkyt L, Morkunas G and Åkerblom G 2005 Indoor radon in the karst region of Lithuania Radioact. Environ. 7 807–12

[24] Stanifer S, Hoover AG, Rademacher K, Rayens MK, Haneberg W and Hahn EJ 2022 Citizen science approach to home radon testing, environmental health literacy and efficacy Citizen Science: Theory and Practice vol 7 26 36845873 10.5334/cstp.472 PMC9949773

[25] Maeng S, Han SY and Lee SH 2019 Analysis of radon depth profile in soil air after a rainfall by using diffusion model Nucl. Eng. Technol. 51 2013–7

[26] Winkler R, Ruckerbauer F and Bunzl K 2001 Radon concentration in soil gas: a comparison of the variability resulting from different methods, spatial heterogeneity and seasonal fluctuations Sci. Total Environ. 272 273–82 10.1016/s0048-9697(01)00704-5 11379922

[27] Bunzl K, Ruckerbauer F and Winkler R 1998 Temporal and small-scale spatial variability of 222Rn gas in a soil with a high gravel content Sci. Total Environ. 220 157–66 9810724 10.1016/s0048-9697(98)00256-3

[28] Washington JW and Rose AW 1992 Temporal variability of radon concentration in the interstitial gas of soils in Pennsylvania J. Geophys. Res: Solid Earth 97 9145–59

[29] Research Wolfram, Inc. 2022 Mathematica 13.2 edn (Champaign, Illinois: Wolfram Research, Inc.) (available at: www.wolfram.com/mathematica)

[30] Weiss A (ed) 2001 Topographic position and landforms analysis Poster Presentation, ESRI User Conf. (San Diego, CA)

[31] Hahn EJ, Wiggins AT, Rademacher K, Butler KM, Huntington-Moskos L and Rayens MK 2019 FRESH: long-term outcomes of a randomized trial to reduce radon and tobacco smoke in the home Prev. Chronic. Dis 16 E127–E 10.5888/pcd16.180634 PMC6745895 31517597

[32] Brookins DG 1992 Background radiation in the Albuquerque, New Mexico, U.S.A., area Environ. Geol. Water Sci. 19 11–15

[33] Symonds P, Rees D, Daraktchieva Z, McColl N, Bradley J, Hamilton I and Davies M 2019 Home energy efficiency and radon: an observational study Indoor Air 29 854–64 31127966 10.1111/ina.12575 PMC6772076

[34] Barros-Dios JM, Ruano-Ravina A, Gastelu-Iturri J and Figueiras A 2007 Factors underlying residential radon concentration: results from Galicia, Spain Environ. Res. 103 185–90 10.1016/j.envres.2006.04.008 16729995

[35] Yazzie SA, Davis S, Seixas N and Yost MG 2020 Assessing the impact of housing features and environmental factors on home indoor radon concentration levels on the Navajo nation Int. J. Environ. Res. Public Health 17 2813 32325838 10.3390/ijerph17082813 PMC7215699

[36] Simms JA, Pearson DD, Cholowsky NL, Irvine JL, Nielsen ME, Jacques WR, Taron JM, Peters CE, Carlson LE and Goodarzi AA 2021 Younger North Americans are exposed to more radon gas due to occupancy biases within the residential built environment Sci. Rep. 11 6724 10.1038/s41598-021-86096-3 PMC7990966 33762674

[37] Durrani S and Badr I 1995 Geostatistically controlled field study of radon levels and the analysis of their spatial variation Radiat. Meas 25 565–72

[38] Oliver M and Khayrat A 2001 A geostatistical investigation of the spatial variation of radon in soil Comput. Geosci 27 939–57

[39] Zhu H, Charlet J and Poffijn A 2001 Radon risk mapping in southern Belgium: an application of geostatistical and GIS techniques Sci. Total Environ. 272 203–10 10.1016/s0048-9697(01)00693-3 11379911

[40] Fanshawe TR and Diggle PJ 2012 Bivariate geostatistical modelling: a review and an application to spatial variation in radon concentrations Environ. Ecol. Stat 19 139–60

[41] Loffredo F, Scala A, Adinolfi GM, Savino F and Quarto M 2020 A new geostatistical tool for the analysis of the geographical variability of the indoor radon activity Nukleonika 65 99–104

[42] Mulla D and McBratney AB 2002 Soil spatial variability Soil Physics Companion (Boca Raton, FL: CRC Press) p 343–73

[43] Spasić D and Gulan L 2022 High indoor radon case study: influence of meteorological parameters and indication of radon prone area Atmosphere 13 2120

[44] Xie D, Liao M, Wang H and Kearfott KJ 2017 A study of diurnal and short-term variations of indoor radon concentrations at the University of Michigan, USA and their correlations with environmental factors Indoor Built Environ. 26 1051–61

[45] Chen C, Thomas DM and Green RE 1995 Modeling of radon transport in unsaturated soil J. Geophys. Res: Solid Earth 100 15517–25

[46] Kitto M 2005 Interrelationship of indoor radon concentrations, soil-gas flux, and meteorological parameters J. Radioanal. Nucl. Chem. 264 381–5

[47] Keskikuru T, Kokotti H, Lammi S and Kalliokoski P 2001 Effect of various factors on the rate of radon entry into two different types of houses Build. Environ 36 1091–8

[48] Xie D, Liao M and Kearfott KJ 2015 Influence of environmental factors on indoor radon concentration levels in the basement and ground floor of a building–A case study Radiat. Meas 82 52–58

[49] Mentes G and Eper-Pápai I 2015 Investigation of temperature and barometric pressure variation effects on radon concentration in the Sopronbánfalva Geodynamic Observatory, Hungary J. Environ. Radioact. 149 64–72 26207821 10.1016/j.jenvrad.2015.07.015

[50] Belete GD and Shiferaw AM 2022 A review of studies on the seasonal variation of indoor radon-222 concentration Oncol. Rev 16 10570 10.3389/or.2022.10570 PMC9756844 36531161

[51] Kamalakar D, Vinutha P, Kaliprasad C and Narayana Y 2022 Seasonal variation of indoor radon, thoron and their progeny in Belagavi district of Karnataka, India Environ. Monit. Assess. 194 310 35353285 10.1007/s10661-022-09931-8

[52] Rey JF, Goyette S, Gandolla M, Palacios M, Barazza F and Goyette Pernot J 2022 Long-term impacts of weather conditions on indoor radon concentration measurements in Switzerland Atmosphere 13 92

[53] Kellenbenz KR and Shakya KM 2021 Spatial and temporal variations in indoor radon concentrations in Pennsylvania, USA from 1988 to 2018 J. Environ. Radioact. 233 106594 10.1016/j.jenvrad.2021.106594 33798813

[54] Bossew P and Lettner H 2007 Investigations on indoor radon in Austria, Part 1: seasonality of indoor radon concentration J. Environ. Radioact. 98 329–45 17707559 10.1016/j.jenvrad.2007.06.006

[55] Environmental Protection Agency US (EPA) 2016 A citizen’s guide to radon: the guide to protecting yourself and your family from radon Contract No.: EPA402/K-12/002

[56] Mose DG, Mushrush GW, Chrosniak CE and Morgan W 1991 Seasonal indoor radon variations related to precipitation Environ. Mol. Mutagen. 17 223–30 2050129 10.1002/em.2850170402

[57] Mose DG, Mushrush GW and Saiway G (eds) 2010 Summer indoor radon exceeds winter indoor radon Proc. Annual Int. Conf. on Soils, Sediments, Water and Energy

[58] Arvela H, Holmgren O and Hanninen P 2016 Effect of soil moisture on seasonal variation in indoor radon concentration: modelling and measurements in 326 Finnish houses Radiat. Prot. Dosim. 168 277–90 10.1093/rpd/ncv182 PMC4884879 25899611

[59] Groves-Kirkby C, Denman A, Crockett R, Phillips P and Gillmore G 2006 Identification of tidal and climatic influences within domestic radon time-series from Northampton shire, UK Sci. Total Environ. 367 191–202 10.1016/j.scitotenv.2005.11.019 16406058

[60] Sundal AV, Valen V, Soldal O and Strand T 2008 The influence of meteorological parameters on soil radon levels in permeable glacial sediments Sci. Total Environ. 389 418–28 17931690 10.1016/j.scitotenv.2007.09.001

[61] Environmental Law Institute 2012 Radon in homes: strengthening state policy to reduce risk and save lives Research Report (Washington, DC)

[62] U.S. Environmental Protection Agency 2022 The National Radon Action Plan 2021–2015: Eliminating preventable lung cancer from radon in the United States by expanding protections for all communities and buildings

[63] Szabo Z, dePaul VT, Fischer JM, Kraemer TF and Jacobsen E 2012 Occurrence and geochemistry of radium in water from principal drinking-water aquifer systems of the United States Appl. Geochem. 27 729–52

